# Pathway Analysis of Skin from Psoriasis Patients after Adalimumab Treatment Reveals New Early Events in the Anti-Inflammatory Mechanism of Anti-TNF-α

**DOI:** 10.1371/journal.pone.0167437

**Published:** 2016-12-22

**Authors:** Ane Langkilde, Lene C. Olsen, Pål Sætrom, Finn Drabløs, Søren Besenbacher, Line Raaby, Claus Johansen, Lars Iversen

**Affiliations:** 1 Department of Dermatology, Aarhus University Hospital, Aarhus C, Denmark; 2 Department of Cancer Research and Molecular Medicine, Faculty of Medicine, Norwegian University of Science and Technology (NTNU), Trondheim, NORWAY; 3 Department of Computer and Information Science, Faculty of Information Technology, Mathematics and Electrical Engineering, Norwegian University of Science and Technology (NTNU), Trondheim, NORWAY; 4 Bioinformatics Research Centre,Aarhus University, Aarhus C, Denmark; University of Alabama at Birmingham, UNITED STATES

## Abstract

Psoriasis is a chronic cutaneous inflammatory disease. The immunopathogenesis is a complex interplay between T cells, dendritic cells and the epidermis in which T cells and dendritic cells maintain skin inflammation. Anti-tumour necrosis factor (anti-TNF)-α agents have been approved for therapeutic use across a range of inflammatory disorders including psoriasis, but the anti-inflammatory mechanisms of anti-TNF-α in lesional psoriatic skin are not fully understood. We investigated early events in skin from psoriasis patients after treatment with anti-TNF-α antibodies by use of bioinformatics tools. We used the Human Gene 1.0 ST Array to analyse gene expression in punch biopsies taken from psoriatic patients before and also 4 and 14 days after initiation of treatment with the anti-TNF-α agent adalimumab. The gene expression was analysed by gene set enrichment analysis using the Functional Annotation Tool from DAVID Bioinformatics Resources. The most enriched pathway was visualised by the Pathview Package on Kyoto Encyclopedia of Genes and Genomes (KEGG) graphs. The analysis revealed new very early events in psoriasis after adalimumab treatment. Some of these events have been described after longer periods of anti-TNF-α treatment when clinical and histological changes appear, suggesting that effects of anti-TNF-α treatment on gene expression appear very early before clinical and histological changes. Combining microarray data on biopsies from psoriasis patients with pathway analysis allowed us to integrate *in vitro* findings into the identification of mechanisms that may be important *in vivo*. Furthermore, these results may reflect primary effect of anti-TNF-α treatment in contrast to studies of gene expression changes following clinical and histological changes, which may reflect secondary changes correlated to the healing of the skin.

## Introduction

The skin harbors multiple systems that eg. encompasses the skin immunesytem in order to protect the body and maintain local homeostasis [1–3]. Psoriasis is a chronic cutaneous inflammatory disease affecting 2–3% of the population [4]. Its immunopathogenesis has been described as a cross-talk between T cells, dendritic cells (DCs) and the epidermis [5, 6]. A complex interplay generates the inflammatory process, and it was previously described that T cells and DCs maintain skin inflammation [7]. Studies of skin samples from patients treated with anti-tumour necrosis factor (anti-TNF)-α agents have confirmed that DCs and T cells play a role in the pathogenesis of psoriasis, and anti-TNF-α has been shown to regulate both Th17 responses and IL-23 and Th17 cytokine expression in lesional psoriatic skin [8, 9].

Many gene expression studies of the *in vivo* effect of anti-TNF-α has been conducted two weeks or later after treatment start where the histological and clinical changes start appearing. Findings at these time points reveal genes that may be important in the pathogenesis of psoriasis as well as direct or indirect targets of anti-TNF-α agents [10, 11].

Anti-TNF-α agents have been approved for therapeutic use across a range of inflammatory disorders, including Crohn’s disease, rheumatoid arthritis, spondyloarthritis and psoriasis [12–16]. Despite the fact that >500,000 patients worldwide have been treated with anti-TNF-α drugs like infliximab, adalimumab and etanercept, the anti-inflammatory mechanisms of these agents in lesional psoriatic skin are not fully understood [8]. TNF-α achieves its effect through a complicated interplay of both cell differentiation and expression of inflammatory genes [17, 18]. *In vitro* models may not be able to fully explain the mode of action of TNF-α in gene regulation in inflammatory diseases, and *in vivo* studies therefore still seem warranted [8].

Inhibition of TNF-α is known to modulate a wide panel of genes involved in immune and inflammatory responses. Some of these genes, including IL-8, IL-1β, CXCL10, and CXCL11 represent direct targets of TNF-α [19–21], whereas others are the result of an indirect effect that reflects changes in the cell and cytokine network occurring within the target tissues [8–10, 22, 23]

High-throughput technologies like gene expression arrays provide a fundamental starting point in the identification of mechanistic causes and regulatory signalling pathways. One standard method involves identifying differentially expressed genes (DEGs). However, this method offers only limited information regarding the biological function of the differentially expressed genes. Another method is gene set enrichment analysis (GSEA) which uses pathway knowledge to detect biologically relevant signals; e.g. the Kyoto Encyclopedia of Genes and Genomes (KEGG) pathways enriched by a set of genes can be identified and visualised and this approach provides a better understanding of relevant DEGs in a given context.

In the present study, we analysed punch biopsies from psoriatic patients before treatment and after adalimumab treatment; untreated nonlesional skin (NL), untreated lesional skin (L0), lesional skin 4 days after treatment start (L4) and lesional skin 14 days after treatment start (L14). We used the Human Gene 1.0 ST Array and the gene expression was analysed by GSEA using the Functional Annotation Tool from DAVID Bioinformatics Resources [24, 25]. The most enriched pathway was visualised using the Pathview Package on KEGG graphs [26].

## Materials and Methods

### Patient material

The patients included were adults (> 18 years) with psoriasis vulgaris who were going to be treated with anti-TNF-α drugs independently of the project. Patients were excluded if they had received local treatment two weeks before inclusion or had received any systemic treatment including UVB 6 weeks prior to the inclusion date or five half-lives of the drug, whichever was longest. At inclusion patients started treatment with adalimumab 80 mg at week 0, 40 mg at week 1 followed by 40 mg every other week. Treatment was started according to local guidelines and independent of this protocol. Before and during treatment, patients were monitored clinically with a Psoriasis Area Severity Index (PASI) score. Clinical evaluation was conducted by a trained dermatologist at the session at which punch biopsies were also taken. The study was approved by the regional ethical committee of Region Midtjylland (approval no. m-20090102) and performed according to the principles of the Declaration of Helsinki. Signed informed consent was obtained from each patient. 4 mm punch biopsies from lesional and nonlesional psoriatic skin were obtained from 10 psoriatic patients using a local anaesthetic containing 1% lidocaine at day 0 (NL and L0), 4 (L4) and 14 (L14) after initiation of adalimumab treatment (Table 1).

**Table 1:** Details on patients included in the study. Paired punch biopsies from ten psoriasis patients were used. Gender, age and Psoriasis Area and Severity Index (PASI) score are given for each patient; mean age = 50.6 years, mean PASI = 21.15. Available biopsy samples (NL, L, Day 4 and Day 14) are indicated with •.

ID	Gender	Age	NL	L	PASI	Day 4	Day 14
A01	m	65	•	•	19.5	•	•
A02	m	59	•	•	12.8	•	•
A03	m	56	•	•	38.6	•	•
A04	m	36	•	•	21	•	•
A05	m	51	•	•	19.8	•	•
A06	m	52	•	•	15.6	•	•
A07	m	35	•	•	20.3	•	•
A08	f	50	•	•	29.6	•	
A09	f	51	•	•	20.8	•	
A10	f	51	•		13.5	•	

### Gene array

Gene expression analysis on the Human Gene 1.0 ST Array (Affymetrix, Inc., Santa Clara, CA, USA) was performed on ten paired punch biopsies from psoriatic patients. A total of 100 ng RNA was labelled with the Ambion WT Expression Kit (Ambion, Carlsbad, CA, USA) according to the manufacturer’s instructions. Samples were hybridised overnight to the GeneChip and scanned using an Affymetrix GCS 3000 7G scanner.

### Data processing

All array data processing and analysis was done in R version 3.1.2 (2014-10-31).

Background correction and normalisation of the raw data and robust multiarray analysis (RMA) were conducted with the Affy package [27] in R language. The Limma package [28] in R language was then applied to analyse the changes in gene expression values, while taking paired samples into account(untreated nonlesional (NL), untreated lesional psoriatic skin (L0), 4 days after adalimumab treatment start (L4) and 14 days after adalimumab treatment start (L14)).

All DEGs were corrected for multiple testing by Benjamini-Hochberg adjustment. However, due to lack of differentially expressed DEGs reaching a level of significance in the untreated lesional versus day 4 contrast, we performed a selection by a cut-off on +/- 0.58-log fold change (logFC) (+/- 1.5 fold changes) irrespective of p-values for further analyses. Probe IDs were converted into gene symbols using the hugene10sttranscriptcluster.db package. The Limma package [28] in R language was applied to quantitatively examine the statistical differences between samples in a Principal Component Analysis (PCA) plot.

To visualise overlap of DEGs between contrasts, we used the Vennerable package. The selected genes were analysed, and a GSEA was performed in each contrast L0 versus NL, L0 versus L4 and L0 versus L14 using the Functional Annotation Tools from DAVID Bioinformatics Resources [24, 25].

Finally, the one pathway that was significantly enriched (p-value ≤ 0.05) by the selected genes was visualised by use of the Pathview package on KEGG pathway graphs (http://www.genome.jp/kegg/) [26].

## Results

### No genes were significantly regulated when comparing lesional psoriatic skin at day 0 and day 4 after adalimumab treatment initiation

**Fig 1:**
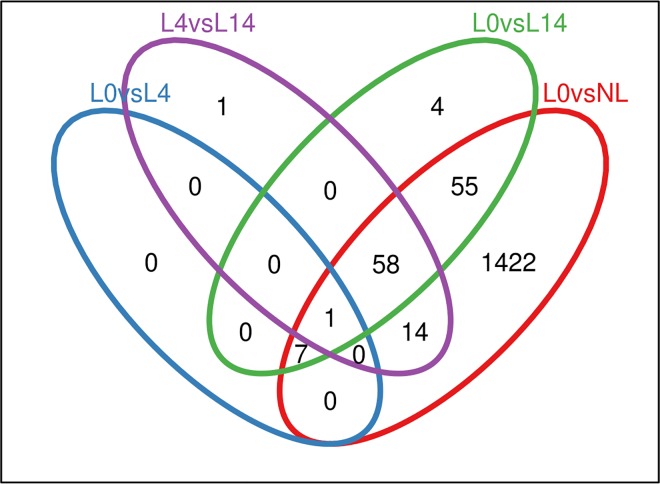
Venn diagram indicating the overlap between differentially expressed genes in each contrast. Blue: lesional untreated versus lesional day 4. Purple: lesional day 4 versus lesional day 14. Green: Lesional untreated versus lesional day 4. Red: Lesional untreated versus nonlesional untreated.

RNA from samples collected from psoriasis patients on days 0 and 4 (n = 10) and on day 14 (n = 7) after adalimumab treatment initiation was analysed using the Affymetrix microarray. We found that 1,610 genes were differentially expressed between NL and L0 when using a +/- 0.58 logFC cut-off. Among the selected genes, 1,598 genes were significantly (adj. p-value < 0.05) differentially expressed in L0 versus NL contrast. Eight genes (none significant) were differentially expressed in L0 versus L4 contrast; 129 genes (9 significantly; adj. p-value < 0.05) were differentially expressed in the L0 versus L14 contrast; and 75 (none significant) genes were differentially expressed in the L4 versus L14 contrast (Fig 1). An overwhelming majority of the genes that were differentially expressed in the L0 versus L4, L0 versus L14, and L4 versus L14 contrasts were also found in the L0 versus NL contrast. This prompted us to examine these overlapping DEGs more closely.

### After adalimumab treatment, the relative gene expression in lesional psoriatic skin changes towards the relative gene expression in nonlesional psoriatic skin

**Fig 2:**
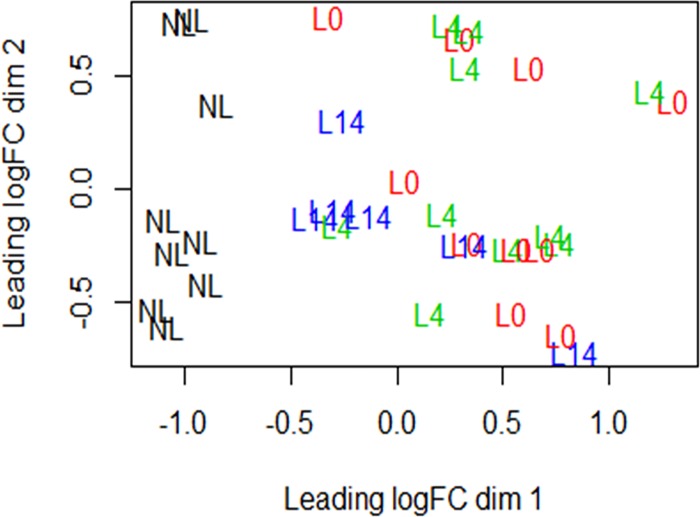
PCA plot of the samples (NL, L0, L4 and L14). The PCA plot shows clear differences between nonlesional skin (NL) and lesional untreated skin (L0). Also lesional skin 14 days after adalimumab treatment initiation (L14) tends to resemble the NL.

A PCA plot showed differences between NL and L0 samples. The L0 and L4 samples appeared similar while L14 samples showed a tendency to approach the expression pattern of NL samples (Fig 2).

**Fig 3:**
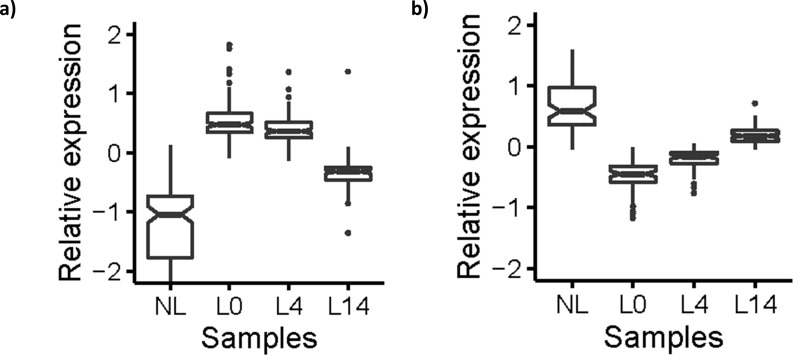
Relative expression of regulated genes in untreated lesional skin. (a) Upregulated and (b) downregulated genes in untreated lesional psoriatic skin. Nonlesional skin (NL), lesional untreated skin (L0), lesional skin on day 4 after adalimumab treatment was initiated (L4) and lesional skin on day 14 after adalimumab treatment was initiated (L14).

We plotted the relative expression of the selected genes, separating genes that were upregulated from those that were downregulated in L0 (Fig 3). Both up- and downregulated genes changed their expression towards the expression seen in nonlesional psoriatic skin. We analysed the genes for enriched gene ontology terms using the DAVID Functional Annotation Tools. The overlapping genes that were upregulated in L0 were enriched in cytokines. There were no significantly enriched terms for the overlapping genes downregulated in L0, although epidermis development was the least non-significant term.

### The KEGG pathway:“Cytokine-cytokine receptor interaction; hsa04060” was significantly enriched after 4 and 14 days

Because no genes were significantly differentially expressed at day 4 after adalimumab treatment initiation, we performed a GSEA of all genes regulated more than +/- 0.58 log FC from each contrast by the David Functional Annotation Tools. We corrected for multiple testing and found four KEGG pathways that were significantly enriched by the genes from the list of 1,610 +/- 0.58 log FC genes (S1 Table) when untreated lesional and nonlesional psoriatic skin was compared. One KEGG pathway, the cytokine-cytokine receptor interaction (hsa04060), was significantly enriched in the genes from the list of eight annotated +/- 0.58 log FC genes when L0 and L4 were compared (adj. p-value = 4.02E-05). It was also significantly enriched in the genes from the list of 129 annotated, +/- 0.58 log fold changed genes (S2 Table) (adj. p-value = 8.90E-03) when comparing L0 with L14. Analysis of the genes involved in the cytokine-cytokine receptor interaction pathway and comparison of untreated lesional with nonlesional psoriatic skin showed that all genes involved were significantly differentially expressed in this contrast as well (S3 Table).

### The KEGG view of the cytokine-cytokine receptor interaction pathway revealed possible early events in the anti-inflammatory mechanism of anti-TNF

**Fig 4:**
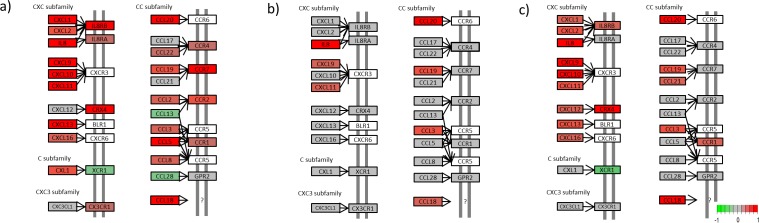
Cytokine-cytokine receptor interaction for the chemokines. (a) Untreated lesional versus nonlesional, (b) Untreated lesional versus 4 days after adalimumab treatment was initiated and (c) Untreated lesional versus 14 days after adalimumab treatment was initiated. (Colours refer to the expression level; green is downregulated, red is upregulated in untreated lesional psoriatic skin, grey is not regulated and white is not included in our data set) (Please find list of gene names in S4 Table).

**Fig 5:**
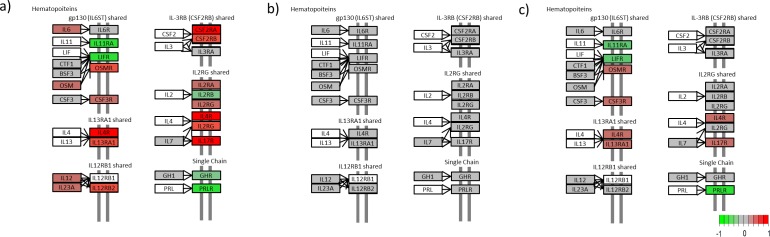
Cytokine-cytokine receptor interaction for the Hematopoiteins. (a) Untreated lesional versus nonlesional, (b) Untreated lesional versus 4 days after adalimumab treatment was initiated and (c) Untreated lesional versus 14 days after adalimumab treatment was initiated. (Colours refer to the expression level; green is downregulated, red is upregulated in untreated lesional psoriatic skin, grey is not regulated and white is not included in our data set) (Please find list of gene names in S4 Table).

**Fig 6:**
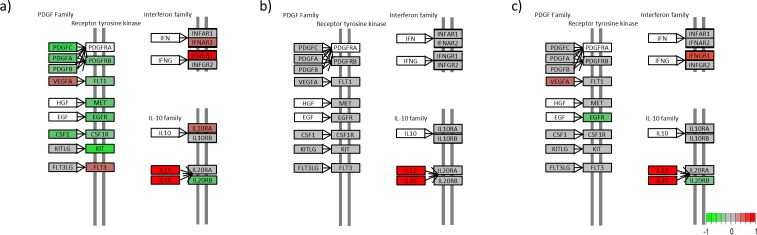
Cytokine-cytokine receptor interaction for the PDGF family, Interferon family and IL-10 family. (a) Untreated lesional versus nonlesional, (b) Untreated lesional versus 4 days after adalimumab treatment was initiated and (c) Untreated lesional versus 14 days after adalimumab treatment was initiated. (Colours refer to the expression level; green is downregulated, red is upregulated in untreated lesional psoriatic skin, grey is not regulated and white is not included in our data set) (Please find list of gene names in S4 Table).

**Fig 7:**
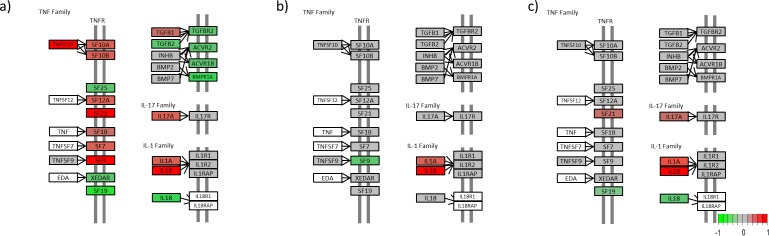
Cytokine-cytokine receptor interaction for the TNF family, IL-17 family and IL-1 family. (a) Untreated lesional versus nonlesional, (b) Untreated lesional versus 4 days after adalimumab treatment was initiated and (c) Untreated lesional versus 14 days after adalimumab treatment was initiated. (Colours refer to the expression level; green is downregulated, red is upregulated in untreated lesional psoriatic skin, grey is not regulated and white is not included in our data set) (Please find list of gene names in S4 Table).

We generated a KEGG view of the most enriched pathway; the cytokine-cytokine receptor interaction pathway (Figs 4–7). The KEGG view makes it possible to follow the gene expression patterns in this pathway over time.

Looking at the regulation of chemokines in the cytokine-cytokine receptor interaction pathway it is clear that 25 chemokines (*IL8*, *IL8RB*, *IL8RA*, *CXCR4*, *CCR1*, *CXCL1*, *CXCL2*, *CXCL9*, *CXCL10*, *CXCL11*, *CXCL13*, *CXCL16*, *CXL1*, *CX3CR1*, *CCL3*, *CCL5*, *CCL8*, *CCL18*, *CCL19*, *CCL20*, *CCL22*, *CCR1*, *CCR2*, *CCR4* and *CCR7*) were upregulated and 3 (*XCR1*, *CCL13* and *CCL28*) were downregulated in L0 compared with NL (Fig 4A). After 4 days of treatment, only seven chemokines (*IL8*, *CXCL9*, *CXCL11*, *CCL3*, *CCL18*, *CCL19* and *CCL20*) showed changes in expression level compared with untreated lesional psoriatic skin (Fig 4B). In Fig 4B, these changes are shown as upregulation in L0 relative to L4, to make them comparable with the plot of L0 vs NL in Fig 4A, but they actually reflect the overall downregulation in L4 relative to L0 following treatment with adalimumab. This will be the case also for the remaining examples of L0 vs L4 or L14, where a depicted upregulation in the figures (to make it comparable to L0 vs NL) is discussed as downregulation following the changes in L4 and L14, and vice versa. After 14 days of treatment, 18 chemokines (*IL8*, *IL8RB*, *CXCR4*, *CCR1*, *CXCL1*, *CXCL2*, *CXCL9*, *CXCL10*, *CXCL11*, *CXCL12*, *CXCL13*, *CXCL16*, *CCL3*, *CCL18*, *CCL19*, *CCL20* and *CCL21*) were downregulated and one chemokine (*XCR1*) was upregulated compared with the starting point; untreated lesional psoriatic skin (Fig 4C).

In the KEGG pathway maps, eight proteins normally classified as cytokines were classified as hematopoiteins. The hematopoiteins showed less regulation overall. Comparison of L0 with NL showed that 14 hematopoiteins (*IL6*, *IL12*, *IL23A*, *IL2RA*, *IL2RG*, *IL4R*, *IL12RB2*, *IL13RA1*, *IL17R*, *OSM*, *OSMR*, *CSF2RA*, *CSF2RB* and *CSF3R*) were upregulated and 5 (*IL11RA*, *IL12RB*, *LIFR*, *GHR* and *PRLR*) were downregulated in untreated lesional psoriatic skin (Fig 5A). After 4 days of treatment, no significant changes in regulation were seen (Fig 5B); but after 14 days of treatment, the expression pattern of this protein family began to resemble the one seen when comparing L0 with NL. Five hematopoiteins (*IL4R*, *IL13RA1*, *IL17R*, *CSF3R* and *OSMR*) were downregulated and 3 (*IL11RA*, *LIFR* and *PRLR*) were upregulated compared with untreated lesional psoriatic skin (Fig 5C).

The plateletderived growth factor (*PDGF*) protein family was mainly downregulated in L0 compared with NL. Ten PDGF proteins (*PDGFA*, *PDGFB*, *PDGFC*, *PDGFRB*, *FLT1*, *MET*, *EGFR*, *CSF1*, *CSF1R* and *KIT*) were downregulated and 2 (*VEGFA* and *FLT3*) were upregulated in L0 compared with NL (Fig 6A). No change in regulation was seen in this protein family after 4 days of treatment (Fig 6B); but after 14 days of treatment, *EGFR* was upregulated and *VEGFA* was downregulated compared with L0 (Fig 6C).

In the interferon family, two proteins (*IFNAR2* and *IFNGR1*) were upregulated in L0 compared with NL (Fig 6A). No change in regulation was detected after 4 days of treatment (Fig 6B); but after 14 days, *IFNGR1* was downregulated compared with L0 (Fig 6C).

In the *IL10* protein family, *IL19*, *IL20* and *IL10RA* were upregulated and *IL20RB* was downregulated in L0 compared with NL (Fig 6A). After 4 days of treatment, *IL19* and *IL20* were downregulated (Fig 6B); and after 14 days of treatment, *IL19* and *IL20* were downregulated and IL20RB was upregulated compared with L0 (Fig 6C).

In the TNF protein family, 9 proteins (*TNFSF10*, *SF1B*, *SF7*, *SF9*, *SF10A*, *SF10B*, *SF12A*, *SF21* and *TGFB1*) were upregulated and 8 (*SF19*, *SF25*, *XEDAR*, *TGFB2*, *TGFB2R*, *ACVR1B*, *ACVR2* and *BMPR1A*) were downregulated in L0 compared with NL (Fig 7A). Only *SF9* was upregulated after 4 days of treatment (Fig 7B), whereas *SF21* was downregulated and *SF19* was upregulated after 14 days of treatment compared with L0 (Fig 7C).

*IL17A* was upregulated in L0 compared with NL (Fig 7A) and downregulated in lesional skin after 14 days of treatment compared with L0 (Fig 7C).

*IL1α* and *IL1β* were upregulated and *IL18* was downregulated in L0 compared with NL (Fig 7A). After 4 days of treatment, *IL1α* and *IL1β* were downregulated (Fig 7B); and after 14 days, *IL1α* and *IL1β* were downregulated and *IL18* was upregulated compared with L0 (Fig 7C).

## Discussion

We analysed gene expression in skin from psoriasis patients at day 0, 4 and 14 after initiation of adalimumab treatment, using a Human Gene 1.0 ST Array. We found 1,598 genes showing significant changes in expression level after adjusting for multiple testing and using a +/- 0.58 logFC cut-off. No genes showed significant changes in regulation 4 days after treatment. This was expected because no histological changes were seen at that stage, and only a few genes have previously been described to be regulated that early [29]. However, when viewed overall, genes that are differentially expressed at day 14 clearly show a trend in the same direction as early as at day 4.

The gene expression data were further analysed using the Functional Annotation Tools from DAVID Bioinformatics Resources. Focusing on pathways enriched by genes showing changes in regulation after 4 and 14 days of adalimumab treatment, we found that the only significantly enriched pathway after treatment was that of cytokine-cytokine receptor interaction. This is in line with earlier findings demonstrating that cytokines are regulated early after adalimumab treatment [29].

We generated a KEGG view of this pathway and this provided a more biologically relevant view on gene expression analysis than the one obtained with analysis of individual genes. The direction of gene regulation in pathways does not need to be the same for all involved genes as signalling pathways will frequently show reciprocal gene regulation [30, 31]. Several examples of this were seen in our data.

The KEGG view of the cytokine-cytokine receptor interaction showed how the signalling pathway changed over time and highlighted genes that may change early after treatment.

After 4 days of adalimumab treatment, 11 cytokines were downregulated and one was upregulated compared with untreated lesional psoriatic skin; i.e., their regulatory pattern was moving closer to that of nonlesional skin. IL-8 was initially upregulated in untreated lesional skin, but then downregulated in lesional skin after 4 days of treatment compared with untreated lesional skin. This is in line with earlier findings that TNFα regulates IL-8 [32]. In an ex vivo study of peripheral blood mononuclear cells, anti-TNF-α therapy has been observed to decrease the production of IL-8 [33]. Levels of serum IL-8 have been shown to be significantly decreased in rheumatoid arthritis (RA) patients on anti-TNF-a therapy [34] and reduced IL-8 production may reflect a significant amelioration in the inflammatory state of RA patients[35]. Furthermore, IL-8 is a keratinocyte-released chemokine known to attract neutrophils and T-cells [32, 36] suggesting a role in the immune response in the skin.

*CXCL9* and *CXCL11* were upregulated in untreated lesional psoriatic skin compared with nonlesional skin, and downregulated after 4 days of treatment compared with untreated lesional psoriatic skin. These proteins are CXCR3 ligands described to attract Th1/Th17-transient cells [37]. Interestingly, cytokines synthesized by Th17 cells stimulate keratinocytes and may be one of the most important drivers of the IL-23/Th-17 axis, which is important in driving keratinocyte hyperproliferation in psoriasis [38, 39]. The early downregulation of the two ligands after treatment suggests that TNF-α inhibition also inhibits Th1/Th17 cells through the CXCL9/CXCL11-CXCR-3 pathway.

*CCL19* and *CCL20* were upregulated in untreated lesional psoriatic skin, but downregulated after 4 days of treatment. The expression of CCL19 and its receptor CCR7 by central memory/naïve T cells and maturing dendritic cells has previously been described to be key event in the migration of these cells to the lymph nodes. The presence of lymphoid-like tissue has been observed in the dermis of psoriatic skin notes and have previously been described as important in the early event of decreasing inflammation in response to TNF-α inhibition [10, 11]. Bosè *et al*., (2013) analysed nine psoriasis patients after TNF- α inhibition and correlated gene expression fold change in lesional skin with the Psoriasis Area and Severity Index score decrease induced by TNF blockade after 4 weeks of treatment. After 4 weeks they showed that genes that were mostly associated with the clinical amelioration were CCR7, its ligand, CCL19, and dendritic cell maturation genes suggesting that inhibition of the CCR7/ CCL19 axis is a critical event for the clinical remission of psoriasis in response to TNF-α inhibition [10]. Goldminz *et al*., (2015) compared mRNA expression after two weeks of adalimumab treatment in nine patients (all responders) with mRNA expression in 12 methotrexate treated patients (all responders) [11]. They showed CCL20 downregulation after two weeks of adalimumab treatment and after 16 weeks of methotrexate treatment [11]. The fact that we found regulation of the same genes suggest that the effect of anti-TNF-α may be initiated as early as four days after treatment start.

The *CCL3*, *IL19* and *IL20* genes have been described to be upregulated in untreated lesional psoriatic skin, [40–42] and they were downregulated 4 days after adalimumab treatment start. Finally, *IL1α* and *IL1β* were downregulated after 4 days of adalimumab treatment start. IL-1β and IL-23 are required for IL-17A production in dermal T cells [43].

These results suggest that anti-TNF-α treatment works on a number of genes in the very early phase after treatment.

After 14 days of adalimumab treatment, the number of regulated chemokines had increased substantially. From the Pathview maps, it was observed that the regulation was changing towards the pattern in nonlesional psoriatic skin. This observation confirms that treated skin started to resemble nonlesional psoriatic skin after 14 days of adalimumab treatment. Interestingly, receptors like *IL8RB* and *CCR1* showed downregulation after 14 days. The ligands of these receptors were downregulated after 4 days, which suggests that ligand downregulation may in some situations entail downregulation of their receptors as well.

Overall, these results suggest that one of the early events in the skin of psoriasis patients after adalimumab treatment is decreased attraction of immune cells by first downregulation of chemokines and then their receptors (e.g. *IL8* after 4 days and *IL8RB* after 14 days). Another early event is downregulation of genes involved in angiogenesis, e.g.*CCL19* and *CCL20* [44] at day 4 after treatment start and *VEGFA* [45] after 14 days.

The decrease in VEGF expression is further supported by another study of the effect of anti-TNF-α treatment in RA patients [46]. One week after patients received anti- TNF-α a decrease in VEGF serum concentration was observed and after two weeks, the concentration was returned to pre-treatment levels. These results correlated with decrease in disease sesverity [46].

After 14 days of adalimumab treatment, we observed regulation of cytokines involved in keratinocyte proliferation like *IL13RA1* [47] and *CXCR4* [48]. This is in line with histological changes observed, i.e. a decrease in epidermal thickness. [49] Furthermore, chemokines that are suggested to regulate the Th17 cell population were downregulated (*CXCL1* [50, 51] and *IL17A* [52]). This supports earlier studies showing that TNF-α inhibition in psoriasis is associated with a substantial Th17 cell-count reduction in the patients’ peripheral blood and that this decrease is significantly associated with an adequate response to biologic therapy [53].

Taken together, our pathway analysis provided a new overview of early biological events in psoriasis after adalimumab treatment. It should be emphasised that only patients undergoing adalimumab treatment have been included in this analysis. Therefore, we cannot tell if the biological events after remission are owing to adalimumab treatment or events seen in general after remission.

The pathway analysis tool is useful for classifying the continually increasing literature that explores the molecular mechanisms of the pathogenesis of psoriasis and remission after treatment. Many *in vitro* experiments have revealed local mechanistic events in simple models containing few cell types or few tissue types, but these experiments may not reflect the real-life biological changes as the interplay between different cell types and tissues is lacking in these models. The pathway analysis tool used in the present paper and its combination with microarray analysis on biopsies from psoriasis patients is a way of integrating earlier *in vitro* findings in the analysis and employing these findings when investigating the mechanisms that are involved *in vivo*. The inclusion of samples taken very early after anti-TNF-α treatment may add further knowledge to the effect of anti-TNF-α treatment. It can be discussed whether the effects seen after histological and clinical changes start appearing, is a primary effect of anti-TNF-α treatment or a secondary effect because of healing of the skin. Our results may better predict the primary effects of anti-TNF-α treatment as no histological of clinical changes in the skin are to be found this early.

Few have investigated gene expression in lesional psoriatic skin as early as four days after treatment start. This is probably due to the fact that it is very difficult to discover events this early as no histological or clinical changes appears and the differences in the gene expression may be very small.

Few have investigated gene expression in lesional psoriatic skin as early as four days after treatment start. It is difficult to discover events this early as no histological or clinical changes has appeared and the differences in the gene expression may be very small.

The approach of integrating the GSEA made us discover possible expression changes four days after treatment start. A number of these changes have been shown in other studies of anti-TNF-α treatment after histological and clinical changes start appearing. Our result suggest that these changes in gene expression may appear as early as four days after treatment start and earlier than the histological and clinical changes start appearing.

## Supporting Information

S1 Table 1,610 +/- 0.58 log FC genes in the comparason of untreated lesional and nonlesional psoriatic skin. Gene expression analysis on the Human Gene 1.0 ST Array was performed on ten paired punch biopsies from psoriatic patients. Data processing and analysis was done in R version 3.1.2 (2014-10-31). Background correction and normalisation of the RMA were conducted with the Affy package and gene expression analysis was conducted with the Limma package. Probe IDs were converted into gene symbols using the hugene10sttranscriptcluster.db package. All DEGs were corrected for multiple testing by Benjamini-Hochberg adjustment and a selection by a cut-off on +/- 0.58-log fold change (logFC) (+/- 1.5 fold changes) irrespective of p-values was performed. Blue: significantly downregulated in lesional psoriatic skin compared with nonlesional psoriatic skin. Red: significantly upregulated in lesional psoriatic skin compared with nonlesional skin. Black: nonsignificantly +/- 0.58 log FC genes.
(XLSX)

S2 Table +/- 0.58 log FC genes in the comparason of lesional psoriatic skin and psoriatic skin 4 days after adalimumab treatment and in the comparason of lesional psoriatic skin and psoriatic skin 14 days after adalimumab treatment. Gene expression analysis on the Human Gene 1.0 ST Array was performed on ten paired punch biopsies from psoriatic patients. Data processing and analysis was done in R version 3.1.2 (2014-10-31). Background correction and normalisation of the RMA were conducted with the Affy package and gene expression analysis was conducted with the Limma package. Probe IDs were converted into gene symbols using the hugene10sttranscriptcluster.db package. All DEGs were corrected for multiple testing by Benjamini-Hochberg adjustment and a selection by a cut-off on +/- 0.58-log fold change (logFC) (+/- 1.5 fold changes) irrespective of p-values was performed. 8 genes +/- 0.58 log FC regulated between lesional psoriatic skin and at day 4 after adalimumab treatment. 129 genes +/- 0.58 log FC regulated between lesional psoriatic skin and at day 14 after adalimumab treatment. Blue: significantly downregulated in lesinal psoriatic skin compared with 14 days after adalimumab treatoment. Red: significantly upregulated in lesional psoriatic skin compared with 14 days after adalimumab treatment. Black: nonsignificantly +/- 0.58 log FC genes.
(XLSX)

S3 Table Genes involved in Cytokine-cytokine receptor pathway. Gene expression analysis on the Human Gene 1.0 ST Array was performed on ten paired punch biopsies from psoriatic patients. Data processing and analysis was done in R version 3.1.2 (2014-10-31). Background correction and normalisation of the RMA were conducted with the Affy package and gene expression analysis was conducted with the Limma package. Probe IDs were converted into gene symbols using the hugene10sttranscriptcluster.db package. All DEGs were corrected for multiple testing by Benjamini-Hochberg adjustment and a selection by a cut-off on +/- 0.58-log fold change (logFC) (+/- 1.5 fold changes) irrespective of p-values was performed. 10 significantly upregulated genes in lesional psoriatic skin compared with 14 days genes—all involved in the Cytokine-cytokine receptor pathway.
(XLSX)

S4 Table List of gene names. Gene expression analysis on the Human Gene 1.0 ST Array was performed on ten paired punch biopsies from psoriatic patients. Probe IDs were converted into gene symbols using the hugene10sttranscriptcluster.db package.
(XLSX)
